# How Important is the Choice of Bandwidth in Kernel Equating?

**DOI:** 10.1177/01466216211040486

**Published:** 2021-10-20

**Authors:** Gabriel Wallin, Jenny Häggström, Marie Wiberg

**Affiliations:** 1 8075 Department of Statistics, USBE, Umeå University.

**Keywords:** kernel equating, continuization, bandwidth selection, evaluation

## Abstract

Kernel equating uses kernel smoothing techniques to continuize the discrete score distributions when equating test scores from an assessment test. The degree of smoothness of the continuous approximations is determined by the bandwidth. Four bandwidth selection methods are currently available for kernel equating, but no thorough comparison has been made between these methods. The overall aim is to compare these four methods together with two additional methods based on cross-validation in a simulation study. Both equivalent and non-equivalent group designs are used and the number of test takers, test length, and score distributions are all varied. The results show that sample size and test length are important factors for equating accuracy and precision. However, all bandwidth selection methods perform similarly with regards to the mean squared error and the differences in terms of equated scores are small, suggesting that the choice of bandwidth is not critical. The different bandwidth selection methods are also illustrated using real testing data from a college admissions test. Practical implications of the results from the simulation study and the empirical study are discussed.

## Introduction

Kernel equating (KE) is an observed-score equating framework aiming at making test scores from standardized tests comparable between administrations (von Davier et al., 2004). Based on the scores from two test administrations, the objective is to find equivalent scores in terms of the latent trait the test is constructed to measure. Following the Braun and Holland (1982) definition of equivalent scores, KE makes use of the equipercentile transformation to equate the test scores. It is functionally composed of two cumulative distribution functions (CDFs), each representing the respective distribution of the test scores to be equated. For the equipercentile transformation to be properly defined, these functions need to be continuous and monotonically increasing. This is generally not true since test scores most often are discrete. For this reason, KE employs smoothing techniques where a, usually Gaussian, kernel function approximates the discrete CDFs with continuous functions. Regardless of the choice of kernel function (e.g. Gaussian, uniform, and logistic) one needs to select a bandwidth which determines the smoothness of the continuous approximations and ultimately, the equated scores. Since undersmoothing results in estimated distributions that suffer from excessive sampling noise and oversmoothed distributions will blur the characteristics of the underlying density, it is of interest to investigate to what extent the choice of bandwidth influences the KE estimator.

The overall aim of this study is to examine if and how the bandwidth choice affects the equated scores, and if specific bandwidth selection methods are more suitable for certain test scenarios. The bandwidth selection in KE is particularly interesting to investigate since it defines the main difference to traditional equating methods. If the influence of the bandwidth on the equated scores is strong, it is important to know which bandwidth selection method to use. If the choice is not sensitive, it could encourage practitioners that are lacking strong theoretical training to consider using KE. In the KE literature to date, four different bandwidth selection methods have been proposed: the penalty method (von Davier et al., 2004), the double smoothing (DS) method (Häggström & Wiberg, 2014), the cross-validation (CV) method (Liang & von Davier, 2014), and the Silverman’s rule of thumb (SRT) method (Andersson & von Davier, 2014). For clarity, the cross-validation method in Liang and von Davier (2014) is hereinafter referred to as the likelihood cross-validation method (LiCV). In Häggström and Wiberg (2014), comparisons between the DS and penalty method showed slight differences in terms of mean squared error (MSE) of the estimated mean of the equated scores. The largest differences were seen for skewed score distributions. They considered symmetric and skewed data, using both the equivalent groups (EG) design and the non-equivalent groups with anchor test (NEAT) design. In Liang and von Davier (2014), comparisons between the LiCV method and the penalty method showed small differences in terms of bias of the density estimate, but the former was the preferred choice for symmetric data. They considered the EG design for both symmetric, skewed, and bimodal data. In Andersson and von Davier (2014), comparisons between the SRT and penalty method showed great similarities in terms of the equated scores. They considered symmetric and skewed data under both the EG and NEAT design. The DS, LiCV, and SRT methods have thus only been compared with the penalty method and never with each other. Furthermore, the previous studies on bandwidth selection in KE have used different data collection designs and evaluation criteria, making it even harder to compare the results between the studies.

Outside the KE literature, leave-one-out cross-validation (LCV) has been widely discussed in density estimation, see for example Jones et al. (1996), Sheather (2004), and Wasserman (2006). LCV is often used as a benchmark method for novel bandwidth selection methods within the kernel density and kernel regression frameworks, see for example Park and Marron (1990) and Häggström and De Luna (2010). Thus, in addition to the four currently available bandwidth selection methods in KE, LCV as well as a penalized LCV are included in the comparison for completeness.

The six bandwidth methods will be evaluated and compared with each other in a simulation study where the test length, number of test takers, and distributions of the test scores are varied for both the EG and NEAT design. All methods will also be illustrated empirically with real test data from a college admissions test.

The rest of the paper is structured as follows. First, a brief review of KE is given, then the six bandwidth selection methods are described. This is followed by the simulation study and the empirical illustration. The paper is concluded with a discussion together with some practical recommendations.

## The Kernel Equating Framework

KE comprises five steps: (1) Presmoothing the score distributions; (2) Estimating the score probabilities; (3) Continuizing the estimated score distributions; (4) Equating; and (5) Evaluating the estimated equating function (e.g., by calculating the standard error of equating [SEE]; von Davier et al. (2004); González and Wiberg (2017)). In this paper, the third step, for which KE offers a unique solution in comparison with other equating methods, will be examined.

The test scores from test forms X and Y are denoted by *X* and *Y*, respectively, with realizations *x*_*j*_, *j* = 1, …, *J* and *y*_*k*_, *k* = 1, …, *K*. The scores *X* and *Y* are viewed as random variables with CDFs *F*_*X*_ (⋅) and *G*_*Y*_ (⋅), respectively. In the NEAT design, anchor scores *A* with realizations *a*_*l*_, *l* = 1, …, *L*, are also measured. The equipercentile transformation *φ*_*Y*_(*x*) that equates test form X to test form Y is defined as

(1) $$y=\varphi_{Y}\left(x\right)=G_{Y}^{-1}\left(F_{X}\left(x\right)\right)$$

To define the KE estimator of *φ*_*Y*_(*x*) in equation (1), we introduce the following notation: Let $\sigma_{X}^{2}$ denote the variance of *X*, *r*_*j*_ =  Pr (*X* = *x*_*j*_|*T*), the *x*_*j*_ score probability on the target population *T*, and *Z* a standard normal random variable. Define $a_{X}^{2}=\sigma_{X}^{2}/\left(\sigma_{X}^{2}+h_{X}^{2}\right)$ , where *h*_*X*_ is the bandwidth of the *X* score kernel density estimate and *μ*_*X*_ = *∑*_*j*_*x*_*j*_*r*_*j*_. Using a Gaussian kernel, the discrete score variable *X* is replaced by *X* (*h*_*X*_) = *a*_*X*_ (*X* + *h*_*X*_*Z*) + (1 − *a*_*X*_)*μ*_*X*_, where *X* (*h*_*X*_) is a continuous random variable constructed to preserve the first two moments of *X*. By letting $r={\left(r_{1},\ldots ,r_{J}\right)}^{⊤}$ and Φ(*z*) denote the standard normal distribution function, it can be shown that the CDF of *X* (*h*_*X*_) is given by

(2) $$\begin{array}{ll} F_{h_{X}}\left(x;r\right) & =\text{Pr}\left(X\left(h_{X}\right)\leq x\right)\\ & =\sum_{j}r_{j}\Phi \left(\frac{x-a_{X}x_{j}-\left(1-a_{X}\right)\mu_{X}}{a_{X}h_{X}}\right). \end{array}$$

The continuized CDF of *Y*, denoted $G_{h_{Y}}\left(y;s\right)$ , is defined analogously using $s={\left(s_{1},\ldots ,s_{K}\right)}^{⊤}$ and *s*_*k*_ =  Pr(*Y* = *y*_*k*_|*T*). By letting $F_{h_{X}}\left(x;\hat{r}\right)={\hat{F}}_{h_{X}}\left(x\right)$ and $G_{h_{Y}}\left(y;\hat{s}\right)={\hat{G}}_{h_{Y}}\left(y\right)$ , the KE estimator of the equipercentile transformation in equation (1) is defined as

(3) $${\hat{\varphi }}_{Y}\left(x\right)={\hat{G}}_{h_{Y}}^{-1}\left({\hat{F}}_{h_{X}}\left(x\right)\right)$$

Equations (2) and (3) show the dependence of the equated scores on the bandwidth through their dependence on the continuized score CDFs. Optimal choices of the bandwidths *h*_*X*_ and *h*_*Y*_ would thus find the members of the family of continuous distributions $\left\{{\hat{F}}_{h_{X}},h_{X}>0\right\}$ and $\left\{{\hat{G}}_{h_{Y}},h_{Y}>0\right\}$ that, composed as ${\hat{\varphi }}_{Y}\left(x\right)$ , yield the best estimator of *φ*_*Y*_(*x*).

The most common evaluation measure of the equating estimator given in equation (3) is the SEE (von Davier et al., 2004). The estimated SEE consists of three components; the Jacobian of the estimated equating transformation, denoted ${\hat{J}}_{\varphi_{Y}}$ , the Jacobian of the design function which maps the (presmoothed) score distributions into **r** and **s**, denoted ${\hat{J}}_{\text{DF}}$ , and a matrix **C** that relates to the covariance matrix of the (presmoothed) score distributions. The SEE of ${\hat{\varphi }}_{Y}\left(x\right)$ is formed by combining these three components and calculating the length of the resulting vector, that is,

(4) $${\text{SEE}}_{Y}\left(x\right)=\left\|{\hat{J}}_{\varphi_{Y}}{\hat{J}}_{DF}C\right\|$$

Another common measure is the Percent Relative Error (PRE; von Davier et al. 2004), which measures the discrepancy between the *p*:th moment of the equated scores and that of the *Y* scores. Letting $\mu_{p}\left(Y\right)=\sum_{k}{\left(y_{k}\right)}^{p}s_{k}$ and ${\hat{\mu }}_{p}\left(\varphi_{Y}\left(X\right)\right)=\sum_{j}{\left({\hat{\varphi }}_{Y}\left(x_{j}\right)\right)}^{p}r_{j}$ , the PRE is defined as

$$\text{PRE}\left(p\right)=100\left(\frac{{\hat{\mu }}_{p}\left(\varphi_{Y}\left(X\right)\right)-\mu_{p}\left(Y\right)}{\mu_{p}\left(Y\right)}\right)$$

## Bandwidth Selection Methods in Kernel Equating

We consider data-driven selection of one bandwidth per test score density estimator. Note, it is also possible to manually select bandwidths that fulfill certain objectives. For example, when selecting a very large bandwidth the KE estimator is similar to the linear equating transformation, and when setting the bandwidths equal to 0.33 the KE estimator will approximate the traditional equipercentile transformation that uses linear interpolation (von Davier et al., 2004). Another possibility is to use adaptive kernels (González & von Davier, 2017) which allow for different bandwidths along the data points. All expressions in this section are in terms of the *X* scores, but expressions for the *Y* scores are analogous.

### The Penalty Method

The most common way of selecting the bandwidth in KE is by minimizing the sum of the squared distances between the estimated score probabilities ${\hat{r}}_{j}$ , *j* = 1, …, *J*, and the estimated density function ${\hat{f}}_{h_{X}}\left(x_{j}\right)$ , *j* = 1, …, *J*, where ${\hat{f}}_{h_{X}}$ denotes the derivative of ${\hat{F}}_{h_{X}}$ . To ensure smoothness, a penalty function can be added to the loss function which prevents the estimated density from exhibiting large fluctuations. The penalty method thus selects the bandwidth that minimizes

(5) $$\text{PEN}\left(h_{X}\right)=\sum_{j}{\left({\hat{r}}_{j}-{\hat{f}}_{h_{X}}\left(x_{j}\right)\right)}^{2}+\kappa \cdot \sum_{j}A_{j}$$

where *A*_*j*_ = 1 if

$$\left[\left({\hat{f}}_{h_{X}}^{\prime }\left(x_{j}-w\right)>0\right)\cap \left({\hat{f}}_{h_{X}}^{\prime }\left(x_{j}+w\right)<0\right)\right]$$

or

$$\left[\left({\hat{f}}_{h_{X}}^{\prime }\left(x_{j}-w\right)<0\right)\cap \left({\hat{f}}_{h_{X}}^{\prime }\left(x_{j}+w\right)>0\right)\right],$$

and *A*_*j*_ = 0 otherwise (Lee & von Davier, 2011; von Davier, 2013). The term *κ* is a weight that determines the size of each penalty, ${\hat{f}}_{h_{X}}^{\prime }\left(x_{j}\right)$ is the derivative of ${\hat{f}}_{h_{X}}\left(x_{j}\right)$ , and *w* is a constant that determines the neighborhood of *x*_*j*_ for which the penalty function will penalize choices of *h*_*X*_ that let ${\hat{f}}_{h_{X}}^{\prime }\left(x_{j}\right)$ change sign. Typically *w* = 0.25 (see e.g., von Davier et al. (2004), Häggström and Wiberg (2014) and Andersson and von Davier (2014)).

### Silverman’s Rule of Thumb

A common loss function when selecting bandwidth in density estimation is the asymptotic mean integrated squared error (AMISE; Jones et al., 1996). For a normally distributed random variable, minimizing the AMISE with respect to the bandwidth results in the approximation known as Silverman’s rule of thumb (Scott, 1992). Andersson and von Davier (2014) implemented this bandwidth for KE which, adjusted for *a*_*X*_, equals

$$\text{SRT}\left(h_{X}\right)=\frac{9\sigma_{X}}{\sqrt{100n_{X}^{2/5}-81}}.$$

### Double Smoothing

DS was introduced by Hall et al. (1992) for nonparametric density estimation and implemented within KE by Häggström and Wiberg (2014). Within KE, the procedure starts by using a large, subjectively chosen pilot bandwidth *q*_*X*_ to estimate $f_{q_{X}}$ at the score values and the values halfway between them, that is, at the points $x^{*}=\left\{x_{l}^{*}\right\}={\left[x_{1},x_{1}+0.5,x_{2},\ldots ,x_{J}-0.5,x_{J}\right]}^{⊤}$ , *l* = 1, …, 2*J* − 1. Next, $f_{h_{X}}$ is estimated at x* using ${\hat{f}}_{q_{X}}$ at the actual score values $x={\left[x_{1},x_{2},\ldots ,x_{J}\right]}^{⊤}$ instead of using the estimated score probabilities ${\hat{r}}_{j}$ . Thus, a DS estimate ${\hat{f}}_{h_{X}}^{*}$ is obtained. The bandwidth that minimizes the sum of the squared difference between the *l*th DS estimate ${\hat{f}}_{h_{X}}^{*}\left(x\right)$ and ${\hat{r}}_{l}^{*}$ is selected, where

$${\hat{f}}_{h_{X}}^{*}\left(x\right)=\sum_{j=1}^{J}{\hat{f}}_{q_{X}}\left(x_{j}\right)\phi \left(\frac{x-{\hat{a}}_{X}x_{j}-\left(1-{\hat{a}}_{X}\right){\hat{\mu }}_{X}}{h_{X}{\hat{a}}_{X}}\right)\frac{1}{h_{X}{\hat{a}}_{X}},$$

*ϕ*(*z*) denotes the standard normal density function,

$${\hat{f}}_{q_{X}}\left(x\right)=\sum_{j=1}^{J}r_{j}\phi \left(\frac{x-{\hat{a}}_{X}^{q_{X}}x_{j}-\left(1-{\hat{a}}_{X}^{q_{X}}\right){\hat{\mu }}_{X}}{q_{X}{\hat{a}}_{X}^{q_{X}}}\right)\frac{1}{q_{X}{\hat{a}}_{X}^{q_{X}}},$$

with ${\hat{a}}_{X}^{q_{X}}=\sqrt{{\hat{\sigma }}_{X}^{2}/\left({\hat{\sigma }}_{X}^{2}+q_{X}^{2}\right)}$ and ${\hat{r}}_{l}^{*}={\hat{r}}_{l+1/2}^{*}$ if *l* is even and ${\hat{r}}_{l}^{*}={\hat{f}}_{h_{X}}^{*}\left(x_{l}^{*}\right)$ if *l* is odd.

The DS criterion can be written as

$$\text{DS}\left(h_{X}\right)=\sum_{l=1}^{2J-1}{\left({\hat{r}}_{l}^{*}-{\hat{f}}_{h_{X}}^{*}\left(x_{l}^{*}\right)\right)}^{2}.$$

### The Likelihood Cross-Validation Method

LiCV applied to KE was suggested by Liang and von Davier (2014), and their method of bandwidth selection starts by randomly splitting the data into two subsamples. The first subsample is used to estimate a set of Gaussian kernel densities,

$${\hat{f}}_{h_{X}}^{\left(1\right)}=\sum_{j}{\hat{r}}_{j}\phi \left(\frac{x-{\hat{a}}_{X}x_{j}-\left(1-{\hat{a}}_{X}\right){\hat{\mu }}_{X}}{{\hat{a}}_{X}h_{X}}\right)$$

for a set of bandwidths *h* = [0.01, 0.02, …, 5], where the “(1)” notation indicates that the quantities are calculated using only the first subsample. The density for each value of *h* is then used as an intensity parameter in a set of Poisson likelihood functions, where the score frequencies are taken from the second subsample. The value of *h* that maximizes the likelihood function is stored. The criterion of the LiCV method can be expressed as

$$\text{LiCV}\left(h_{X}\right)=\underset{h}{\text{max}}L\left(n_{x_{j}};{\hat{f}}_{h_{X}}^{\left(1\right)}\right)=\underset{h}{\text{max}}\prod_{j=1}^{J}\frac{e^{-N_{X}^{\left(1\right)}{\hat{f}}_{h_{X}}^{\left(1\right)}\left(x_{j}\right)}{\left(N_{X}^{\left(1\right)}{\hat{f}}_{h_{X}}^{\left(1\right)}\left(x_{j}\right)\right)}^{n_{x_{j}}^{\left(2\right)}}}{n_{x_{j}}^{\left(2\right)}!}$$

where $N_{X}^{\left(1\right)}$ is the number of test takers in the first subsample, and $n_{x_{j}}^{\left(2\right)}$ is the number of test takers with *X* = *x*_*j*_ in the second subsample. This procedure of randomly splitting the data set and selecting the bandwidth that maximizes the Poisson likelihood function is repeated 1000 times and the median of the resulting 1000 bandwidths is selected as the optimal bandwidth.

### Penalized Leave-One-Out Cross-Validation

There are two objectives when estimating the distribution of *X* (*h*_*X*_); ${\hat{f}}_{h_{X}}$ should both be a good estimate of the true density *f * but also track the shape of the relative score frequencies. Regarding the first objective, Stone (1984) showed that

$$\frac{\int {\left(f\left(x\right)-{\hat{f}}_{h_{CV}}\left(x\right)\right)}^{2}dx}{\underset{h}{\text{inf}}\int {\left(f\left(x\right)-{\hat{f}}_{h}\left(x\right)\right)}^{2}dx}\overset{a˙s}{\to }1$$

where ${\hat{f}}_{h_{CV}}\left(x\right)$ is the kernel density estimator of *f* with bandwidth *h*_*CV*_ selected using LCV. To make sure that the estimated density of *X* (*h*_*X*_) also tracks the estimated probabilities the following criterion, for a Gaussian kernel, can be minimized

(6) $$\text{LCV}\left(h_{X}\right)=\frac{1}{J}\sum_{j=1}^{J}{\left({\hat{r}}_{j}-{\hat{f}}_{h_{X}}^{-j}\left(x_{j}\right)\right)}^{2}$$

where

$${\hat{f}}_{h_{X}}^{-j}\left(x_{j}\right)=\sum_{\begin{array}{ll} l=1 & l\neq j \end{array}}^{J}{\hat{r}}_{l}\phi \left(\frac{x_{j}-{\hat{a}}_{X}x_{l}-\left(1-{\hat{a}}_{X}\right){\hat{\mu }}_{X}}{h_{X}{\hat{a}}_{X}}\right)\frac{1}{h_{X}{\hat{a}}_{X}},$$

is the estimate of *f* (*x*_*j*_) based on the subsample with $\left(x_{j},{\hat{r}}_{j}\right)$ left out. The estimated quantities ${\hat{a}}_{X}$ and ${\hat{\mu }}_{X}$ are based on the full sample. Note that the expression in equation (6) is analogous to the first term in equation (5), the criterion of the penalty method. By the same argument used to motivate the second term of the penalty method, we propose to modify the LCV method by adding the penalty function *A*_*j*_. A penalized LCV criterion is thus defined as

$$\text{PLCV}\left(h_{X}\right)=\frac{1}{J}\sum_{j=1}^{J}{\left({\hat{r}}_{j}-{\hat{f}}_{h_{X}}^{-j}\left(x_{j}\right)\right)}^{2}+\kappa \cdot \sum_{j=1}^{J}A_{j}.$$

## Simulation Study

A simulation study is conducted under both the EG and NEAT design to evaluate 1) how big the differences are between the bandwidths described in the previous section and 2) if any such differences are reflected in the equated scores. Most of the presented results are based on the NEAT design since it is a very common design in practice. Additionally, the EG results are often in line with those of the NEAT design except when indicated.

### Simulation Design

All simulations are repeated with 1000 iterations each, with sample sizes of *n* = {100, 1000, 5000}, test lengths of *J* − 1 = *K* − 1 = {40, 80}, and anchor test lengths of *L* − 1 = {20, 40}. For the two smallest sample sizes, both test lengths {40, 80} are considered in combination with both anchor test lengths {20, 40}, and for *n* = 5000, a test length of 80 together with an anchor test length of 40 is considered. By altering the test lengths in this fashion, it is possible to explore how the equating function is affected by a changing number of observed-score frequencies, both on the main test and on the anchor test. It should be noted that relatively long tests can suffer from other issues as well, like a changing shape of the score distributions and a weaker correlation between the anchor and the test scores. These factors are assumed to be negligible in this study.

Data generation and all computations are performed with the software R (R Core Team, 2018) and the R package **kequate** (Andersson et al., 2013) is used for kernel equating. R code for the simulation study can be obtained from the corresponding author upon request.

The data generating process (DGP) described below was chosen in an attempt to mimic the characteristics of real testing data. The scores of the test takers from population *P* who are given test form X are denoted *X* and the scores of the test takers from population *Q* who are given test form Y are denoted *Y*, where we consider number-correct scoring. In the EG design of this study, the two samples of test takers are only randomly different from each other, that is, *P* = *Q*. In the NEAT design, *P* ≠ *Q* and a population weight of 0.5 is used for the target population, that is, *T* = 0.5*P* + 0.5*Q*. Since previous studies on bandwidth selection in KE have used post-stratification in the NEAT design to form the equipercentile transformation function (von Davier et al., 2004), this is the approach here as well to allow the results to be more easily compared. We now describe the DGP for test form X.

1. Generating true score probabilities *r_j_* =  Pr (*X* = *x_j_*) and *p_jl_* =  Pr (*X* = *x_j_* ∩ *A* = *a_l_*), *j* = 1, …, *J*, *l* = 1, …, *L*.

Generate auxiliary variable(s) according to:

For EG

$$U_{i}∼\text{Beta}\left(\alpha ,\beta \right),$$

And for NEAT

$$\begin{array}{l} U_{i},V_{i}∼\text{Normal copula bivariate distribution with Beta}\left(\alpha \text{, }\beta \right)\\ \;\;\;\text{marginal, correlation set to }\rho \;=0.75\text{ and }i=1,\ldots ,n. \end{array}$$

Then individual scores, to be used for generating *r*_*j*_ and *p*_*jl*_, are calculated by rounding the auxiliary variable(s) times the test length to the nearest integer, $X_{i}^{*}=\left\lfloor \left(J-1\right)U_{i}\right\rceil$ and for NEAT also $A_{i}^{*}=\left\lfloor \left(L-1\right)V_{i}\right\rceil ,\;i=1,\ldots ,n.$ We use a combination of floor and ceiling notation to denote rounding to the nearest integer. The floor function of a variable *x*, ⌊*x*⌋, returns the greatest integer less than or equal to *x*, and the ceiling function ⌈*x*⌉ returns the smallest integer greater than or equal to *x*. Under both the EG and NEAT design, the shape parameters for the beta distributions are set to (*α*, *β*) = {(5, 5), (5, 2), (2, 5)} to produce symmetric, negatively skewed and positively skewed score data, respectively. To produce bimodal score data, a mixture of Beta distributions with (*α*, *β*) = (25, 15) and (*α*, *β*) = (15, 25) is used. In the NEAT design, the correlation between the test score and anchor test score is set to *ρ* = 0.75, since it operationally has been standard practice to aim for anchor tests with strong correlation to the total test scores. The R package **copula** (Yan, 2007) is used to generate data from a Normal copula bivariate distribution with Beta marginals.

Let $n_{J}^{*}=\left\{n_{j}^{*}=\sum_{i=1}^{n}I\left(X_{i}^{*}=x_{j}\right)\right\}$ and $n_{JL}^{*}=\left\{n_{jl}^{*}=\sum_{i=1}^{n}I\left(X_{i}^{*}=x_{j}\cap A_{i}^{*}=a_{l}\right)\right\}$ , *j* = 1, …, *J* and *l* = 1, …, *L*. For the EG design, log-linear models regressing $n_{j}^{*}$ on a function of *x*_*j*_ are fitted. The Akaike information criterion (AIC; Akaike, 1998) is used for model selection under the EG design and the Bayesian information criterion (BIC; Schwarz, 1978) is used for model selection under the NEAT design, aiming at parsimonious models. The choice of criteria are based on previous research where the AIC has been shown to be a suitable model-fit measure for univariate log-linear models (Moses & Holland, 2009) and the BIC has been shown to be effective for bivariate smoothing (Moses & Holland, 2010). The fitted values from the AIC/BIC selected models are used as true score probabilities. A similar procedure is used to generate the fitted probabilities under the NEAT design (for details, see Section 11.1 in von Davier et al., 2004). The procedure of using fitted values from estimated log-linear models as true score probabilities follows the approach in both Liang and von Davier (2014) and Häggström and Wiberg (2014). Note that this step is only performed once (per simulation configuration) and the same true probabilities are then used in all simulation iterations.

2. Generating test score frequencies

For each simulation iteration, using the true score probabilities generated in step 1, we generate test score sample frequencies as

$$n_{J}∼\text{Multinomial}\left(n,{\left(r_{1},\ldots ,r_{J}\right)}^{⊤}\right)\;\text{and}\;n_{JL}∼\text{Multinomial}\left(n,{\left(p_{11},\ldots ,p_{JL}\right)}^{⊤}\right)$$

**Figure 1.**
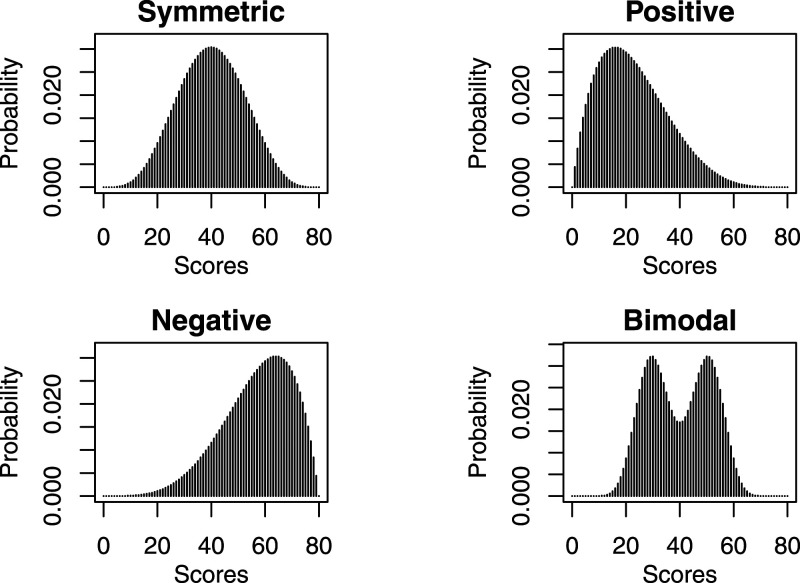
The distributional settings under the EG design in the simulation study.

The DGP for test form Y is analogous to that of test form X with exception that under the NEAT design, the data from population *Q* is shifted by five units along the score axis. This means that for the symmetric, negative, and bimodal distributional scenarios the test form taken by the *Q* sample is more difficult than that taken by the *P* sample, and vice versa for the positive distributional scenario. Figure 1 illustrates the score distributions considered under the EG design.

For the EG design, the log-linear models in step 1 in the DGP preserved the first two and three moments, respectively, of the *X* and *Y* scores for the symmetric and skewed distributions, and the first three moments for the bimodal distributions. For the NEAT design, the models preserved the first four moments of the *X*, *Y*, and *A* scores, respectively, and the first and second cross-moment for the symmetric and negatively skewed distributions. The models for the positively skewed and bimodal distributions preserved the first two moments of the *X*, *Y*, and *A* scores, and the first cross-moment. An alternative would have been to preserve the same number of moments for the different data collection designs. However, we have chosen to use the best fitting model according to the AIC/BIC which we believe to be a better reflection of equating done in practice. Lastly, since it is very common to presmooth the score distributions, the sampled data from step 2 were presmoothed in each simulation iteration using the same models as the ones used to generate the true score probabilities. Finally, note that since the populations of test takers have been created using two random samples from the beta distribution, the identity function is not the true equating function for any of the considered data collection designs.

### Evaluation Criteria

To evaluate the equating results generated by the different bandwidth methods, a comparison between the distribution of ${\hat{\varphi }}_{Y}\left(X\right)$ , the estimated KE transformation evaluated at the discrete *X* score points, and the distribution of *Y* was made for each KE estimator. Let *μ*_*Y*_ = *∑*_*k*_*y*_*k*_*s*_*k*_ , ${\hat{\mu }}_{Y}=\sum_{j}{\hat{\varphi }}_{Y}\left(x_{j}\right)r_{j}$ and let ${\hat{\mu }}_{Y}^{\left(g\right)}=\sum_{j}{\hat{\varphi }}_{Y}^{\left(g\right)}\left(x_{j}\right)r_{j}$ denote the estimator of the mean based on the *g*th replicate. The MSE of ${\hat{\mu }}_{Y}$ over 1000 replications was calculated as

$$\text{MSE}\left({\hat{\mu }}_{Y}\right)={\left[\frac{1}{1000}\sum_{g=1}^{1000}\left({\hat{\mu }}_{Y}^{\left(g\right)}-\mu_{Y}\right)\right]}^{2}+\frac{1}{1000-1}\sum_{g=1}^{1000}{\left[{\hat{\mu }}_{Y}^{\left(g\right)}-\frac{1}{1000}\sum_{g=1}^{1000}{\hat{\mu }}_{Y}^{\left(g\right)}\right]}^{2}$$

Letting ${\bar{\varphi }}_{Y}\left(x_{j}\right)=\frac{1}{1000}\sum_{g=1}^{1000}{\hat{\varphi }}_{Y}^{\left(g\right)}\left(x_{j}\right)$ , the standard error (SE) of ${\hat{\varphi }}_{Y}\left(x_{j}\right)$ was calculated as

$$\text{SE}\left({\hat{\varphi }}_{Y}\left(x_{j}\right)\right)=\sqrt{\frac{1}{1000-1}\sum_{g=1}^{1000}{\left({\hat{\varphi }}_{Y}^{\left(g\right)}\left(x_{j}\right)-{\bar{\varphi }}_{Y}\left(x_{j}\right)\right)}^{2}}$$

For both the MSE and SE, the corrected sample standard deviation formula is applied, for which the squared distances are divided by *G* − 1 = 1000 − 1. For each method and scenario, the PRE of the first 10 moments were also calculated. Furthermore, the mean equating transformation of the 1000 replicates was calculated for every estimator together with the difference that matters (DTM; Dorans & Feigenbaum, 1994) which is referring to all differences larger than half a raw score unit.

Lastly, to judge the validity of the analytical SEEs in equation (4), bootstrap standard errors were calculated under the EG design with symmetric data in an additional simulation. Here, the sample size was *n* = 10,000, the test length was 40 and no presmoothing was conducted. 1000 bootstrap samples were drawn from each data set.

### Simulation Results

The mean of the bandwidths for each scenario and method were calculated and are found in the supplemental material. Generally speaking, for a given scenario, the bandwidth methods result in very different bandwidths. For example, for the symmetric scenario under the EG design, using 80 items and a sample size of 100, the smallest mean bandwidth for the *X* scores is 0.34 (LCV) and the largest mean bandwidth equals 4.84 (SRT). For the negatively skewed data under the NEAT design with 1000 test takers, 80 items and 20 anchor items, the mean bandwidths for the *X* scores were 0.59 (Penalty), 2.96 (SRT), 0.57 (DS), 3.09 (LiCV), 0.32 (LCV), and 1.05 (PLCV). This kind of spread between the different bandwidth selection methods were typical for all scenarios, see the supplemental material for the full table. The variances of the bandwidths were also calculated and the differences between the methods were mostly small. Generally, the variances of the SRT and PLCV methods were the largest under the EG design and the variance of the LiCV method were the largest under the NEAT design. The LCV method had the lowest variance for every scenario and data design.

Table 1 shows the MSE of ${\hat{\mu }}_{Y}$ for the symmetrical distribution scenario under the NEAT design. For a given sample size the MSE increases when the test length increases, and for a given test length, the MSE decreases when the sample size increases. For these sample size and test length effects, the MSE is decreasing by more when the sample size grows from 100 to 1000 than what it is increasing when the number of items grows from 40 to 80. Furthermore, when the anchor test length increases from 20 to 40 items, the MSE decreases for all sample sizes and both data collection designs. For a sample size of 1000, the MSE is more than halved when the anchor test length is doubled. Notably, when the number of test takers equals 5000, the test length equals 80, and the number of anchor items equal 40, the MSE is down to the same size as for the scenario with 1000 test takers, 40 items, and 20 anchor items. For the other distributional scenarios, the results are in line with those of the symmetric distributional scenario. However, the MSE is generally smaller regardless of distribution under the NEAT design compared to the EG design. The MSE is smallest when the data are bimodal, with the overall smallest MSE under the NEAT design with bimodal data, a sample of 1000 test takers and 40 items. The full table can be viewed in the supplemental material.

**Table 1.** The MSEs for all symmetric distributional scenarios considered under the NEAT design. The asterisk (*) indicates that the number of anchor items equals 40, otherwise they equal 20.

Distribution	Design	Penalty	SRT	DS	LiCV	LCV	PLCV
Symmetric	*n* = 100, *J* − 1 = 40	0.329	0.326	0.328	0.326	0.329	0.327
	*n* = 100, *J* − 1 = 80	1.638	1.624	1.638	1.627	1.638	1.637
	*n* = 1000, *J* − 1 = 40	0.032	0.032	0.032	0.032	0.029	0.032
	*n* = 1000, *J* − 1 = 80	0.111	0.111	0.111	0.111	0.111	0.111
	*n* = 100, *J* − 1 = 80*	1.015	1.024	1.023	1.025	1.021	1.029
	*n* = 1000, *J* − 1 = 80*	0.046	0.046	0.046	0.046	0.046	0.046
	*n* = 5000, *J* − 1 = 80*	0.031	0.031	0.031	0.031	0.030	0.031

**Figure 2.**
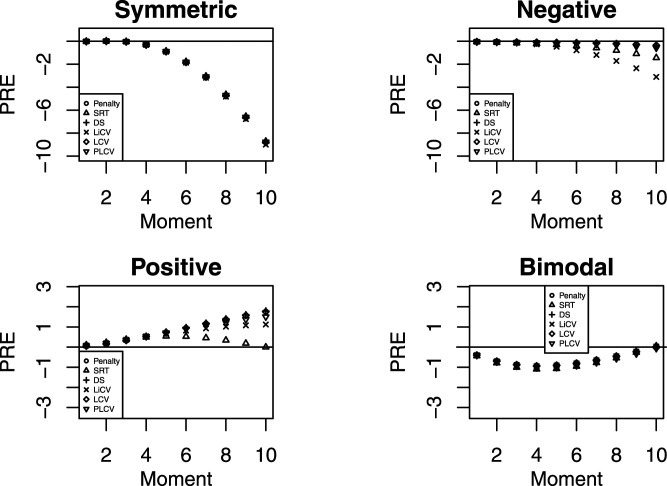
The PRE for every KE estimator under the NEAT design with a sample size of 1000, a test length of 80 and an anchor test length of 20.

Figure 2 shows the performance of each KE estimator in terms of PRE. The results are displayed for the NEAT design using a sample size of 1000 test takers, a test length of 80, and an anchor test length of 20. The PRE for all other sample sizes, test lengths and data collection designs are available and can be found in the supplemental material. For the symmetric distribution, the PREs are very similar and by far the largest in magnitude. For the negatively skewed distributions, the LiCV method is clearly outperformed by the other methods with the SRT method being second worst. In contrast, with positively skewed distributions the LiCV and SRT instead achieve the best results. For the bimodal setting, the differences between the methods are small but with the SRT method performing slightly worse in the mid-range of the scores. The difference in PRE for the other scenarios are generally small. However, the KE estimator using the SRT method is among the worst under the EG design regardless of sample size, test length, and distributional scenario. It is also among the worst for all test lengths and distributional scenarios under the NEAT design when the sample size is small (*n* = 100). It is possible that the relative weak performance of the SRT method is due to its underlying normality assumption which is unrealistic for most test data, including those generated in this simulation study.

**Figure 3.**
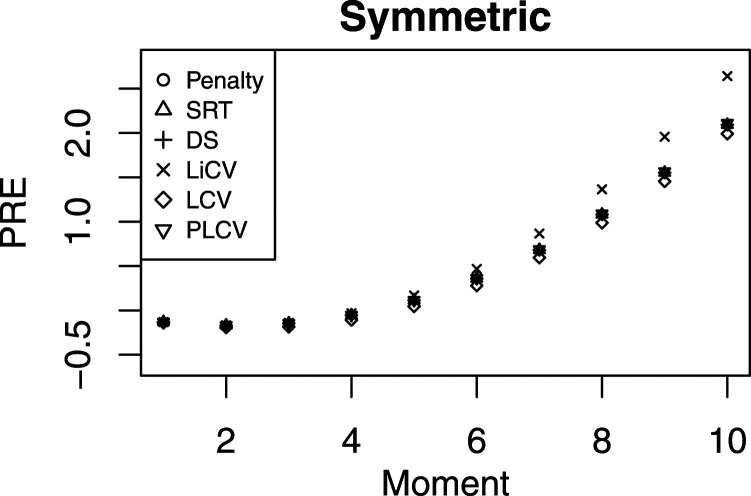
The PRE for every KE estimator for symmetric test score distributions under the NEAT design with a sample size of 5000, a test length of 80, and an anchor length of 40.

In Figure 3, the PRE for the symmetric scenario under the NEAT design is presented when the number of test takers is 5000 per test group, the tests consist of 80 items, and the anchor test length equals 40. As in Figure 2, the differences between the KE estimators are small. In the four last moments, there is a visible, although small, difference between the LiCV-based estimator and the other estimators. It is also notable that the absolute magnitudes of the PREs are considerably smaller compared to when the number of test takers is 1000 and the anchor length is 20. It suggests that increasing the sample size and the length of the anchor test yields an equating estimator that better approximates the Y score distribution.

**Figure 4.**
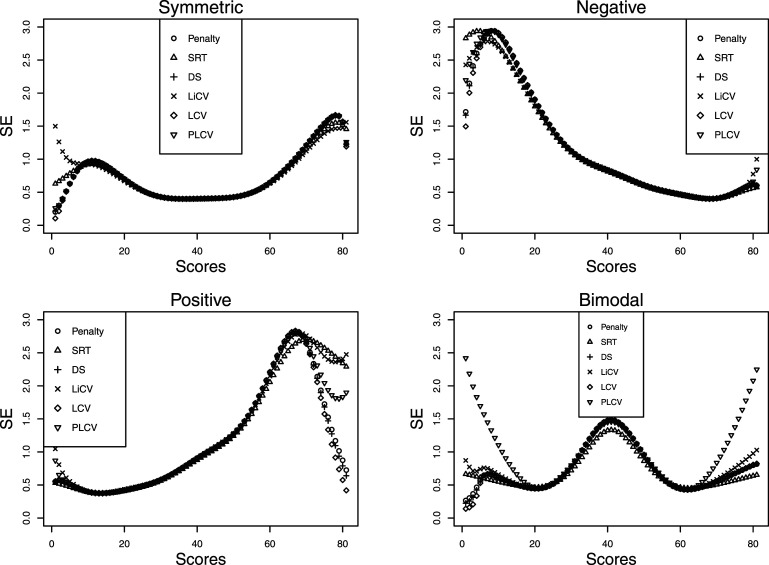
The SE for every KE estimator under the NEAT design with a sample size of 1000, a test length of 80, and an anchor test length of 20.

Figure 4 displays the simulation SEs for the same scenarios as those presented in Figure 2. The estimators are similar in performance but the LiCV method is the worst for the lowest scores in the symmetric setting and the PLCV method the worst in the tails in the bimodal setting. For the negatively skewed data, there are only small differences between each method; however, for positively skewed data the LCV method is superior in the top scores. The distributional scenarios are also reflected in the SE; for the negative skew the SE is substantially higher at the lowest scores, and vice versa for the positive skew.

**Figure 5.**
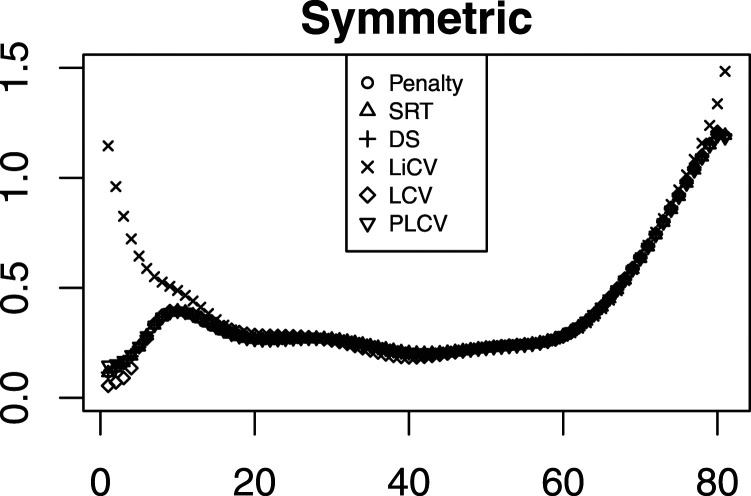
The SE for every KE estimator under the NEAT design with a sample size of 5000, a test length of 80, and an anchor test length of 40.

Figure 5 illustrates the SE for all KE estimators in a similar way as in Figure 4, but with 5000 test takers per group and an anchor test length of 40. As in Figure 4, there are mostly small differences between the KE estimators. The exception is the LiCV-based KE estimator which demonstrates larger SEs in the tails of the score scale, especially for the lowest scores. However, in a practical sense this is often not critical since most sensitive decisions are made at the other end of the score scale. It is interesting to note that there is no apparent difference in the SEs compared to when the sample size is 1000 and the anchor test length is 20, as was evident when comparing the PRE.

**Figure 6.**
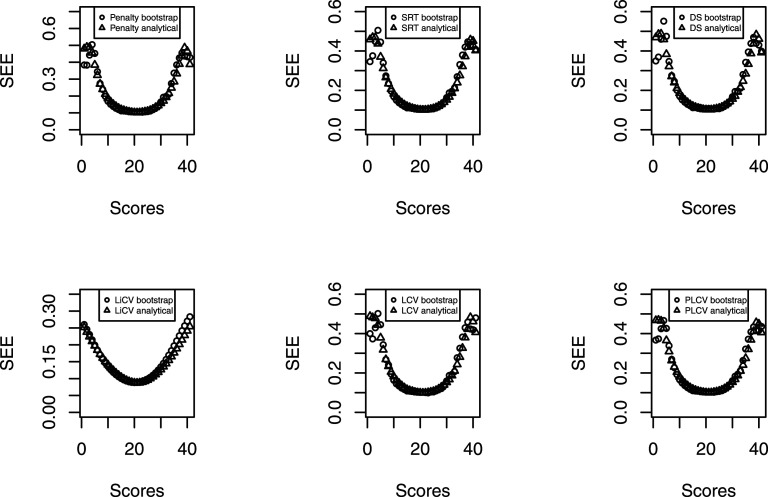
The analytical and bootstrap SEs for every KE estimator under the EG design. The results are based on the symmetric distributional scenario, 10,000 test takers per group and 40 items.

For the estimation of the SE, Figure 6 displays the analytical and bootstrap SE for every KE estimator. There are only slight differences between the analytical and bootstrap SEs for most scores, and differences are seen only at the tails. As expected, the SRT-based KE estimator best manages the tails since it accounts for bandwidth variability in the estimation. However, the DS-based KE estimator also shows a similar pattern even though the analytical SE assumes that the bandwidth is a known constant.

Under the NEAT design with 1000 test takers, 80 items and 20 anchor items, the running times for calculating the bandwidth were 0.17 seconds (Penalty), 0.02 seconds (SRT), 0.03 seconds (DS), 5.13 minutes (LiCV), 0.20 seconds (LCV), and 0.28 seconds (PLCV). The relative performance were similar with 100 and 5000 test takers, and for the EG design. The LiCV clearly deviates because of its vast amount of calculations.

## Empirical Illustration

**Figure 7.**
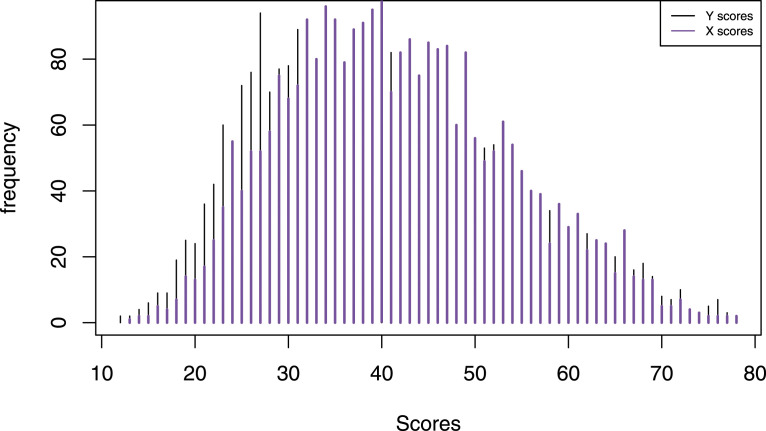
The score distributions for the SweSAT data, with the scores from the spring administration being represented as X scores, and the scores from the autumn administration on the previous year as Y scores.

The Swedish Scholastic Aptitude Test (SweSAT) is a large-scale standardized test used in the admission process to Swedish universities. It is a paper and pencil test that is given twice per year and consists of a quantitative and verbal section, each containing 80 items. The sections are equated separately. To illustrate the KE estimator for different bandwidth selection methods, we equated the quantitative section of the SweSAT using two consecutive administrations. The total sample consisted of 5609 test takers of which 2826 took the spring administration (test form X) and 2783 took the fall administration the year before (test form Y). The mean *X* score was 41.68 with standard deviation 32.46, and the mean *Y* score was 39.89 with standard deviation 29.16. The score distributions are both positively skewed, as can be seen in Figure 7. In practice, SweSAT employs the NEAT design since the assumptions underlying the EG design have been shown to be unfulfilled (Lyrén & Hambleton, 2011). This empirical illustration therefore uses the NEAT design as well. We applied post-stratification with a population weight reflecting the relative group sizes, and presmoothed the samples with log-linear models using the BIC measure to evaluate the goodness-of-fit. This resulted in models that preserved the first 4 moments of the marginal distributions of *X*/*Y* and *A*, respectively, and the first cross-moment of *X*/*Y* and *A*. The SEE and PRE for the first five moments were used to evaluate the equating results.

### Empirical Illustration Results

In the upper part of Table 2, the resulting bandwidths for each method are presented. They clearly differ between each other, with the LCV selecting the smallest bandwidths and the SRT method the largest. The penalty method and the DS method selected very similar bandwidths, and the PLCV method selected bandwidths about twice the size of those selected by LCV. These findings are in line with those from the simulation study. By a graphical inspection, it was seen that the kernel density estimate using the LCV bandwidths resulted in a density with extensive fluctuations. The penalty function in the PLCV method has therefore been activated several times.

**Table 2.** The selected bandwidths under the NEAT design together with the PRE of each KE estimator.

Bandwidth	Penalty	SRT	DS	LiCV	LCV	PLCV
*h*_*X*_	0.73	2.25	0.75	1.75	0.34	0.67
*h*_*Y*_	0.71	2.42	0.74	1.32	0.34	0.65
Moment						
1	0.000	0.000	0.000	0.000	0.000	0.000
2	0.000	0.000	0.000	0.001	−0.003	0.000
3	0.000	0.000	0.000	0.003	−0.009	0.000
4	0.000	0.001	0.000	0.006	−0.012	0.000
5	0.001	0.005	0.001	0.009	−0.027	0.001

In the lower part of Table 2, the PRE of the five first moments are presented for all KE estimators. The PRE is small regardless of bandwidth selection method, but using the penalty, DS, and PLCV methods result in an equating transformation that best preserves the five first moment of the Y score distribution.

**Figure 8.**
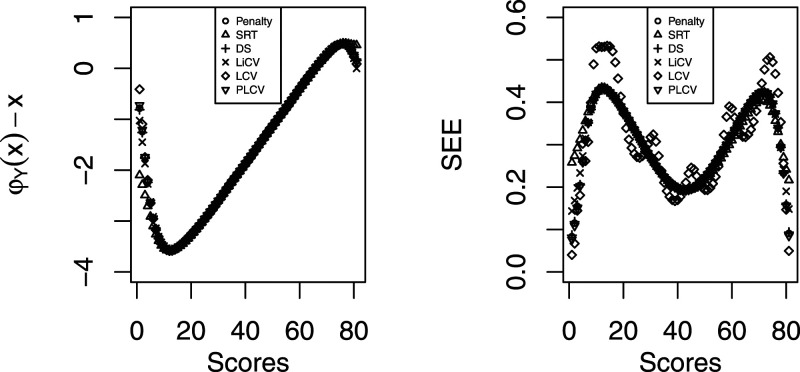
Left panel: The difference between the equated and raw scores for each KE estimator using the SweSAT data. Right panel: The SEE for each KE estimator using the SweSAT data.

In the left panel of Figure 8, the difference between the equated scores and the raw scores are displayed for each KE estimator. The estimators produce very similar results over large parts of the score range, with visible differences only in the tails of the score scale.

In the right panel of Figure 8, the SEE of the KE estimators are shown. Again it is in the tails of the score scale where the differences are most evident. The SEE for all estimators is the largest in the tails, explained by the fact that there are fewer test takers with extreme scores. The PLCV-based estimator has some of the smallest SEEs for the top scores, and the LCV-based estimator has the highest peaks in the tails of the score range.

## Discussion

The overall aim of this study was to compare different bandwidth selection methods in KE, since it is well known that the choice of bandwidth is an essential part of kernel density estimation (Sheather, 2004). Thus, it was important to investigate to what extent the bandwidth has an influence on the equating transformation and if that differed depending on the method used.

The results indicate that the KE estimator is, at least to some extent, insensitive to the choice of bandwidth. Although the selected bandwidths differ between the evaluated methods, the differences in the subsequent equating are small regardless of score distributional shape, sample size, and test length. However, the listed factors are affecting the equating error and variance for every evaluated method. These findings are in line with previous studies (Andersson & von Davier, 2014; Häggström & Wiberg, 2014; Liang & von Davier, 2014) which all found only small differences between their proposed methods and the penalty method. The differences seen in our study were particularly small in terms of MSE. This might be explained by the fact that the MSE is not able to compare the KE estimators over the whole score scale, but only on how well the first two moments of the equated scores correspond to the score distribution that the equated scores attempt to estimate. The results also showed that the MSE under the NEAT design were about half the size compared to that under the EG design, which is in line with the results of Häggström and Wiberg (2014). We believe the reason for this is that the generated test groups were quite similar, although non-equivalent, as you would expect to see in a real testing situation. With an anchor that correlated strongly with the test scores, the variance of the equating transformation should decrease. Since the MSE for the most part was constituted by the variance, the MSE should thus be lower. We also compared every KE estimator with respect to the mean of the equated scores for every score point, and for the most part only small differences were found. However, the results showed that the bandwidth methods sometimes produced equated scores that were larger than a DTM.

The simulation results also showed that the analytical SEE got very close to the bootstrap SE regardless of bandwidth method but with a systematic error at the tails, the SRT method exempted. This means that the variability introduced by the bandwidth choice is not taken into account. For future research, it is thus of importance to derive accurate SEE formulas for all data-driven bandwidth methods.

In terms of PRE, the largest differences between the estimators were seen for the higher moments. At the same time, it should be noted that there are no clear guidelines from previous studies on how many higher moments that are meaningful to compare, or when the magnitude of the PRE indicates a poor equating estimate. Generally, the simulation study showed that shorter tests with more test takers result in smaller PREs and SEs, under both the EG and NEAT design. The promising performance of the LiCV method under the EG design seen in Liang and von Davier (2014) could thus not be repeated under the NEAT design. Moreover, the LiCV method took, by far, the longest time to compute which is not surprising since the procedure has to be repeated 1000 times. It is possible that the LiCV could be calculated using fewer iterations without losing its quality of performance, but this is left for future research.

In order to analyze the influence of bandwidth selection on KE, other factors such as log-linear model specification and the choice of kernel function were purposely marginalized. Since KE involves five steps that affect the equated scores, the simulation study cannot be viewed as exhaustive. One limitation is that we only investigated the impact of the bandwidth on the KE transformation using post-stratification equating. Although it would be of interest to examine the bandwidth impact using chained KE, we do not expect large differences since other studies have showed that post-stratification and chained KE usually give similar results. Future research should also investigate bandwidth selection for item response theory KE.

To conclude, the findings of this paper show that the choice of bandwidth in KE is not crucial in terms of equated scores, but that there still are factors that could make some of the bandwidth methods more appealing. The penalty, DS, and PLCV methods are most robust to changes in test length, number of test takers, and score distributions. They are also quick to compute and could thus be recommended in practice. Our findings also makes the difference between equipercentile equating, linear equating and KE smaller, since the traditional approaches are part of KE as a special case. Practitioners can therefore make use of the flexibility of KE without having to be too concerned about the choice of smoothing parameter. Since previous research has reached similar conclusions regarding the choice of kernel function, the critical part of equating instead seems to lie at the first step, the log-linear presmoothing. The results of this study therefore gives further strength to the view of KE as a family of equating methods that both incorporates traditional and modern equating methods, rather than being a completely new method of equating. KE therefore offers an easy way to both equate test forms and perform sensitivity analysis of the results, by making it possible to compare not only a very smooth equating function or traditional equating, but every possible equating function in between these two modes.

## Supplemental Material

sj-pdf-1-apm-10.1177_01466216211040486 – Supplemental Material for How Important is the Choice of Bandwidth in Kernel Equating? Click here for additional data file. Supplemental Material, sj-pdf-1-apm-10.1177_01466216211040486 for How Important is the Choice of Bandwidth in Kernel Equating? by Gabriel Wallin, Jenny Häggström and Marie Wiberg in Applied Psychological Measurement
