# Supplementary material for: How Important is the Choice of Bandwidth in Kernel Equating?
Source: Appl Psychol Meas. 2021 Oct 20;45(7-8):518–35. doi: 10.1177/01466216211040486 (PMC8640352; doi:10.1177/01466216211040486)
Supplement: sj-pdf-1-apm-10.1177_01466216211040486 – Supplemental Material for How Important is the Choice of Bandwidth in Kernel Equating? [file sj-pdf-1-apm-10.1177_01466216211040486.pdf]

Appendix A - The NEAT distributions in the simulation study

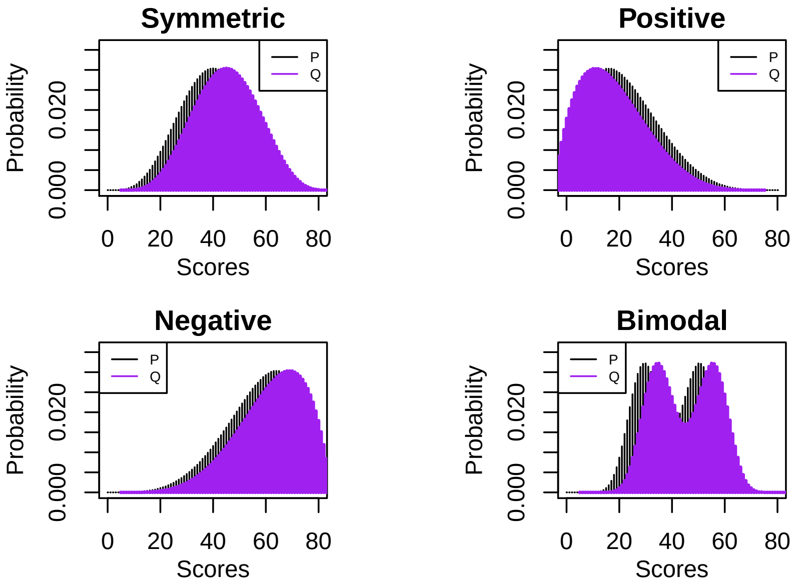

Table B1

The mean bandwidths for the different designs, with the mean bandwidths for Y within parentheses.

		EG						NEAT					
Distribution	Scenario	Penalty	SRT	DS	LiCV	LCV	PLCV	Penalty	SRT	DS	LiCV	LCV	PLCV
Symmetric	$n=100, k=40$	1.11	2.41	0.71	0.44	0.36	0.63	1.61	1.57	1.52	1.27	1.28	1.28
		(1.17)	(2.32)	(0.70)	(0.43)	(0.37)	(0.62)	(1.95)	(0.93)	(1.82)	(0.80)	(0.67)	(2.68)
	$n=100, k=80$	0.71	4.84	0.71	0.42	0.34	0.67	0.69	4.68	0.65	4.70	0.34	0.73
		(0.72)	(4.66)	(0.72)	(0.41)	(0.34)	(0.68)	(0.65)	(5.06)	(0.64)	(4.37)	(0.34)	(0.75)
	$n=1000, k=40$	0.64	1.43	0.64	0.51	0.37	0.88	0.70	1.40	0.69	3.55	0.37	0.67
		(0.70)	(1.40)	(0.70)	(0.59)	(0.37)	(0.68)	(0.63)	(1.37)	(0.66)	(4.06)	(0.37)	(0.65)
	$n=1000, k=80$	0.68	2.86	0.66	0.42	0.34	0.84	0.72	2.80	0.72	3.87	0.34	0.71
		(0.72)	(2.79)	(0.72)	(0.46)	(0.34)	(0.70)	(0.70)	(2.74)	(0.70)	(4.31)	(0.34)	(0.69)
Pos. skewed	$n=100, k=40$	0.53	2.71	0.53	0.47	0.35	0.74	0.55	2.56	0.55	2.74	0.35	0.83
		(0.63)	(2.38)	(0.55)	(0.58)	(0.36)	(0.93)	(0.64)	(2.66)	(0.64)	(2.65)	(0.36)	(0.75)
	$n=100, k=80$	0.55	5.45	0.54	0.43	0.32	0.74	0.563	5.091	0.558	2.847	0.322	0.804
		(0.87)	(4.75)	(0.55)	(0.45)	(0.33)	(0.95)	(0.597)	(5.312)	(0.600)	(1.827)	(0.327)	(0.789)
	$n=1000, k=40$	0.54	1.48	0.54	0.73	0.35	0.72	0.54	1.48	0.54	0.57	0.35	0.76
		(0.56)	(1.42)	(0.54)	(0.46)	(0.35)	(0.73)	(0.66)	(1.46)	(0.66)	(3.25)	(0.36)	(0.65)
	$n=1000, k=80$	0.55	2.95	0.55	0.40	0.32	0.72	0.60	2.96	0.55	1.80	0.32	1.12
		(0.57)	(2.85)	(0.56)	(0.42)	(0.32)	(0.79)	(0.64)	(2.91)	(0.55)	(1.75)	(0.33)	(1.11)
Neg. skewed	$n=100, k=40$	0.53	2.71	0.53	0.47	0.35	0.75	0.64	2.40	0.54	2.97	0.35	1.32
		(0.63)	(2.38)	(0.55)	(0.59)	(0.36)	(0.95)	(0.64)	(2.69)	(0.51)	(2.30)	(0.34)	(1.38)
	$n=100, k=80$	0.55	5.43	0.54	0.43	0.32	0.75	0.76	4.84	0.55	4.50	0.32	1.20
		(0.85)	(4.74)	(0.55)	(0.45)	(0.33)	(0.94)	(0.76)	(5.41)	(0.55)	(4.43)	(0.32)	(1.15)
	$n=1000, k=40$	0.54	1.47	0.54	0.72	0.35	0.70	0.58	1.48	0.55	1.68	0.35	1.34
		(0.56)	(1.42)	(0.54)	(0.47)	(0.35)	(0.75)	(0.59)	(1.46)	(0.54)	(0.69)	(0.36)	(1.37)
	$n=1000, k=80$	0.55	2.95	0.55	0.40	0.32	0.73	0.59	2.96	0.57	3.09	0.32	1.05
		(0.57)	(2.84)	(0.56)	(0.42)	(0.32)	(0.80)	(0.65)	(2.91)	(0.57)	(1.43)	(0.33)	(1.12)
Bimodal	$n=100, k=40$	0.67	2.24	0.62	0.51	0.37	0.74	4.40	0.98	3.41	0.84	0.70	2.42
		(0.66)	(2.23)	(0.61)	(0.51)	(0.37)	(0.75)	(4.95)	(0.93)	(3.82)	(0.80)	(0.67)	(2.68)
	$n=100, k=80$	0.70	4.48	0.65	0.44	0.34	0.73	0.81	4.53	0.72	4.97	0.34	0.67
		(0.70)	(4.45)	(0.64)	(0.44)	(0.34)	(0.75)	(0.69)	(4.71)	(0.70)	(5.00)	(0.34)	(0.68)
	$n=1000, k=40$	0.71	1.37	0.70	0.59	0.37	0.63	0.75	1.37	0.78	2.82	0.37	0.60
		(0.70)	(1.41)	(0.70)	(0.60)	(0.37)	(0.66)	(0.65)	(1.36)	(0.68)	(3.18)	(0.37)	(0.60)
	$n=1000, k=80$	0.73	2.75	0.72	0.45	0.34	0.67	0.59	3.51	0.49	0.78	0.31	1.29
		(0.72)	(2.81)	(0.72)	(0.46)	(0.34)	(0.69)	(0.59)	(3.46)	(0.49)	(0.75)	(0.31)	(1.36)

Appendix C - Results from the simulation study

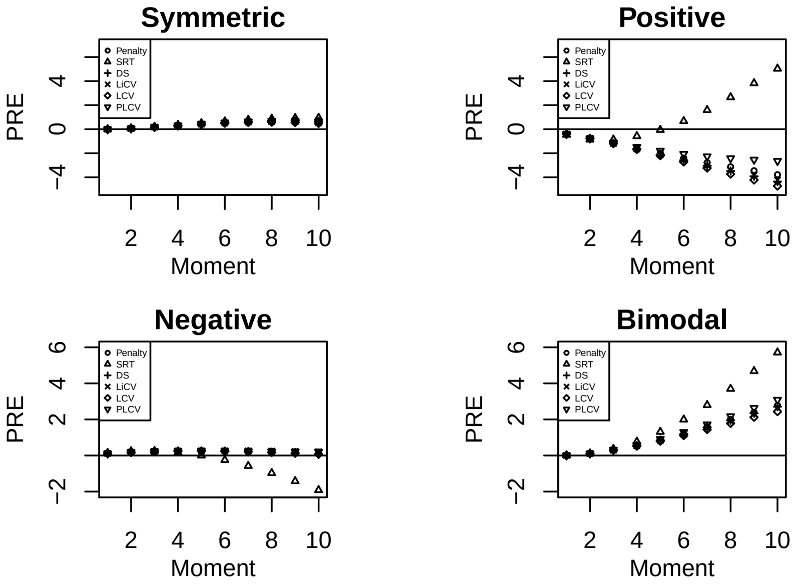

Figure C1: The PRE under the EG design with a sample size of 100 and a test length of 40.

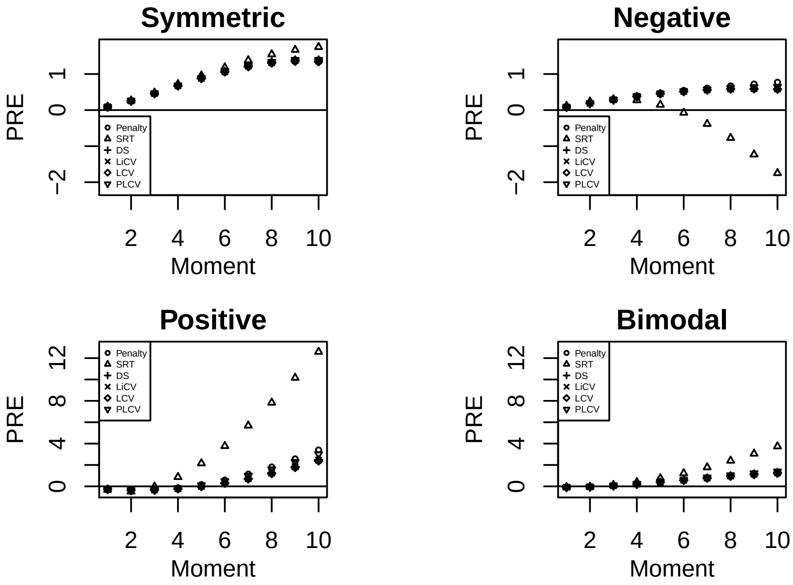

Figure C2: The PRE under the EG design with a sample size of 100 and a test length of 80.

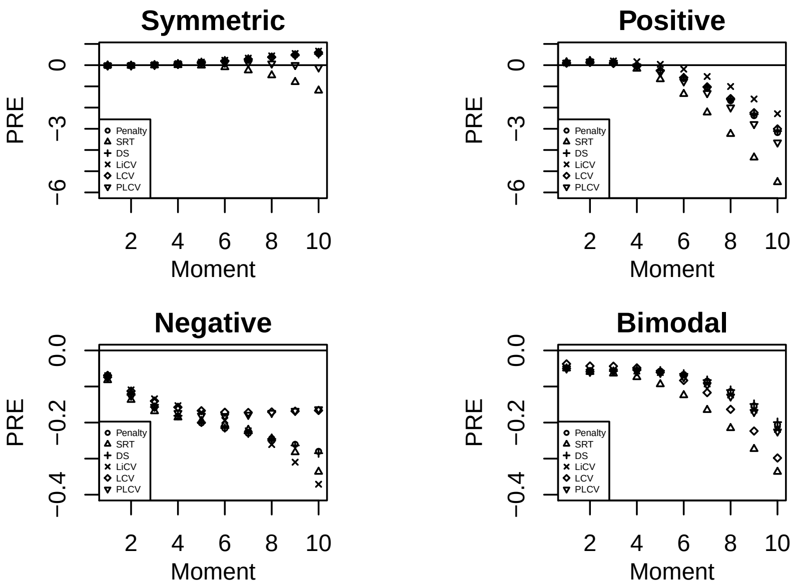

Figure C3: The PRE under the EG design with a sample size of 1000 and a test length of 40.

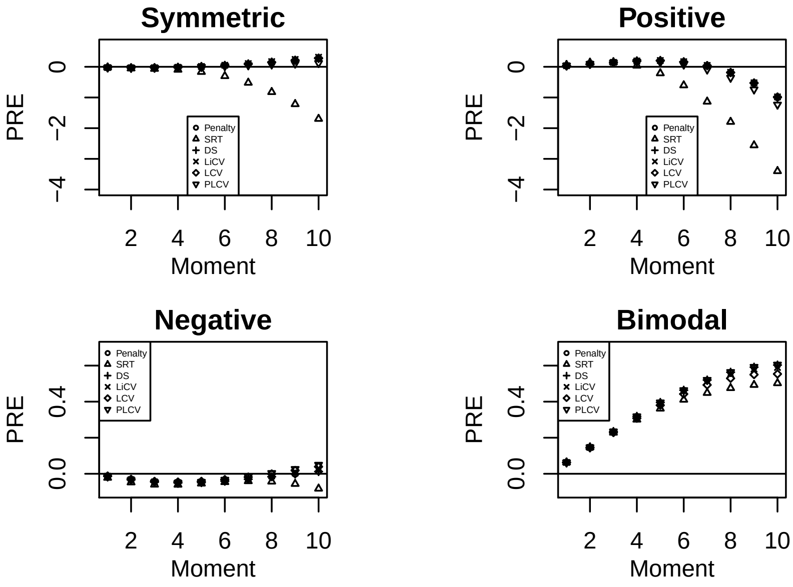

Figure C4: The PRE under the EG design with a sample size of 1000 and a test length of 80.

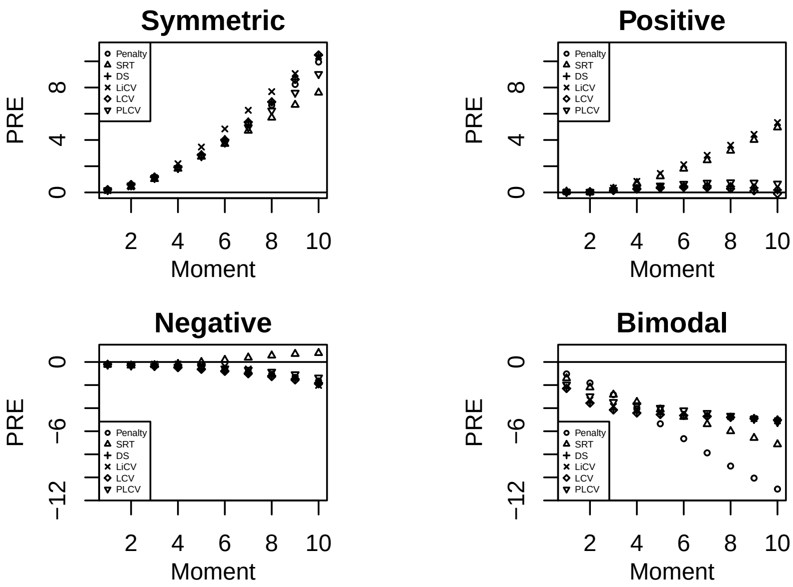

Figure C5: The PRE under the NEAT design with a sample size of 100 and a test length of 40.

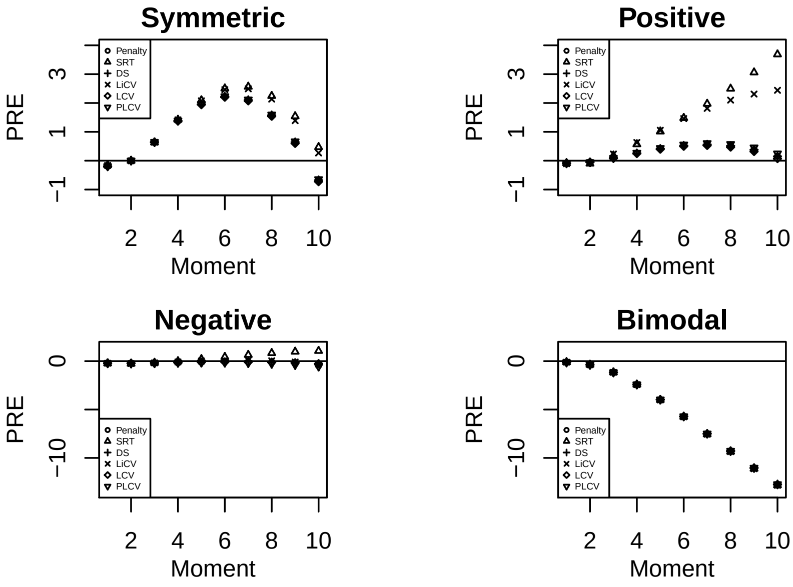

Figure C6: The PRE under the NEAT design with a sample size of 100 and a test length of 80.

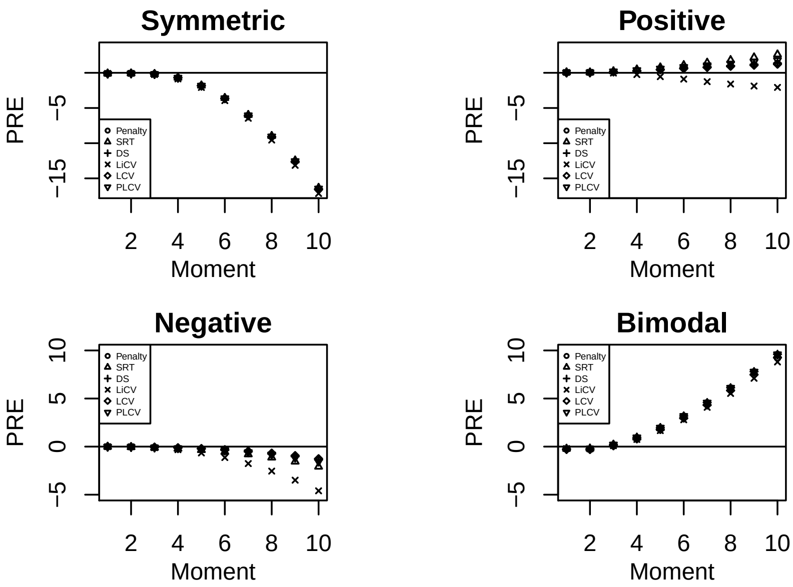

Figure C7: The PRE under the NEAT design with a sample size of 1000 and a test length of 40.

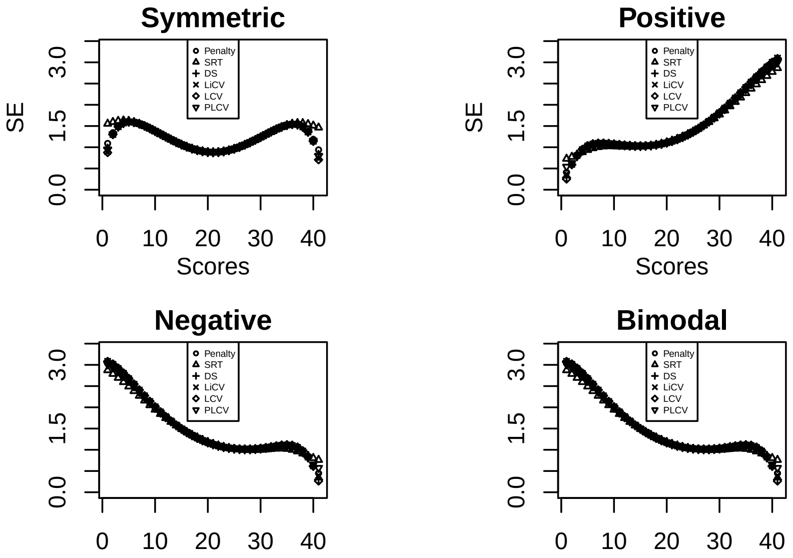

Figure C8: The SE under the EG design with a sample size of 100 and a test length of 40.

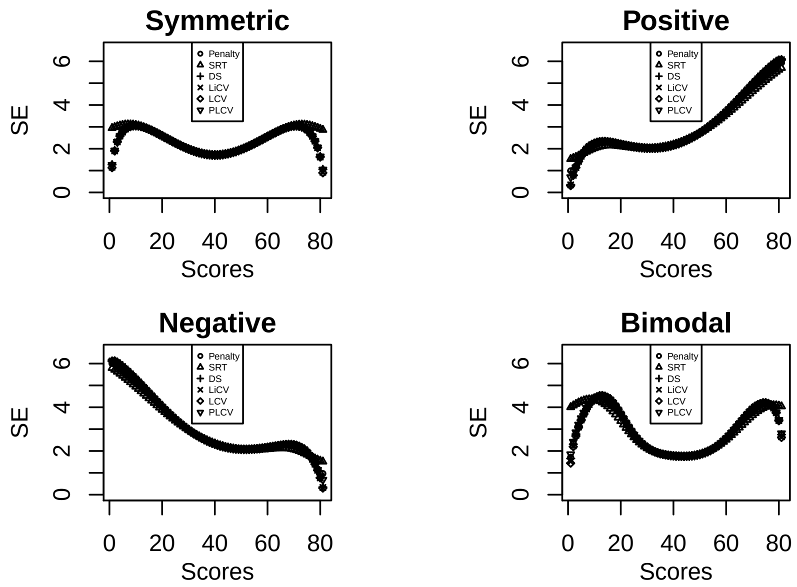

Figure C9: The SE under the EG design with a sample size of 100 and a test length of 80.

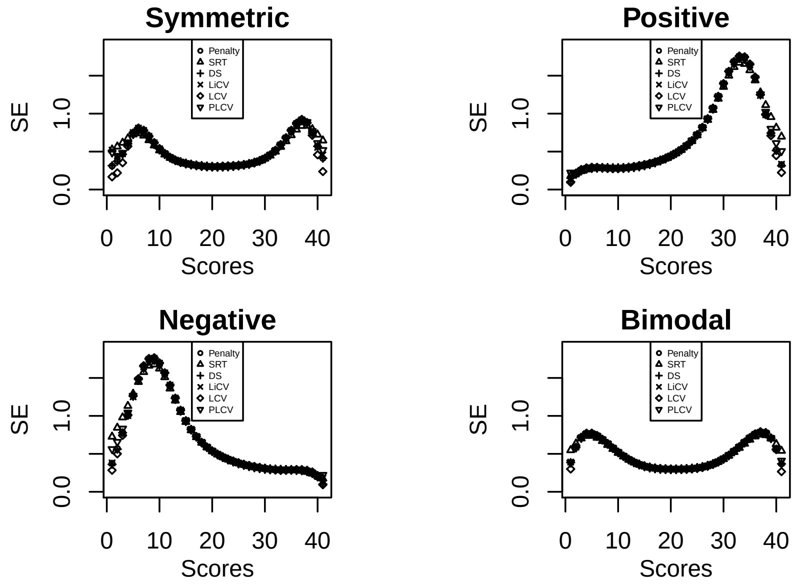

Figure C10: The SE under the EG design with a sample size of 1000 and a test length of 40.

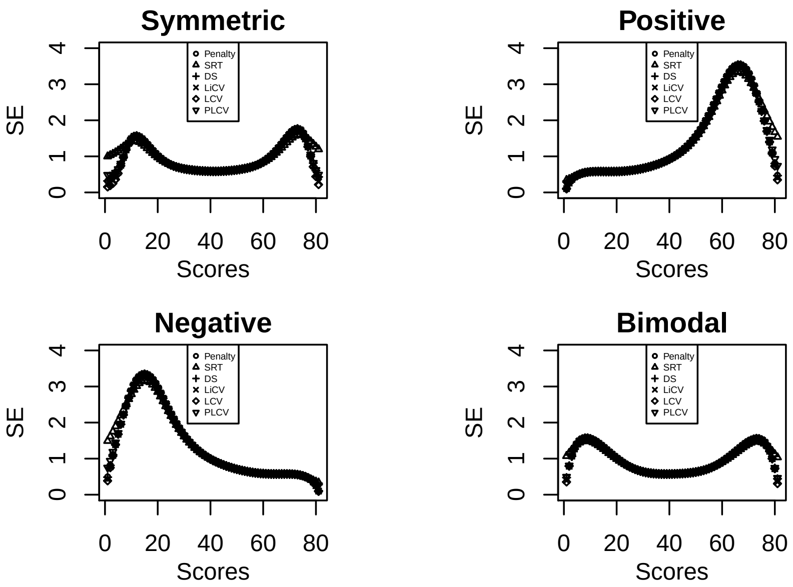

Figure C11: The SE under the EG design with a sample size of 1000 and a test length of 80.

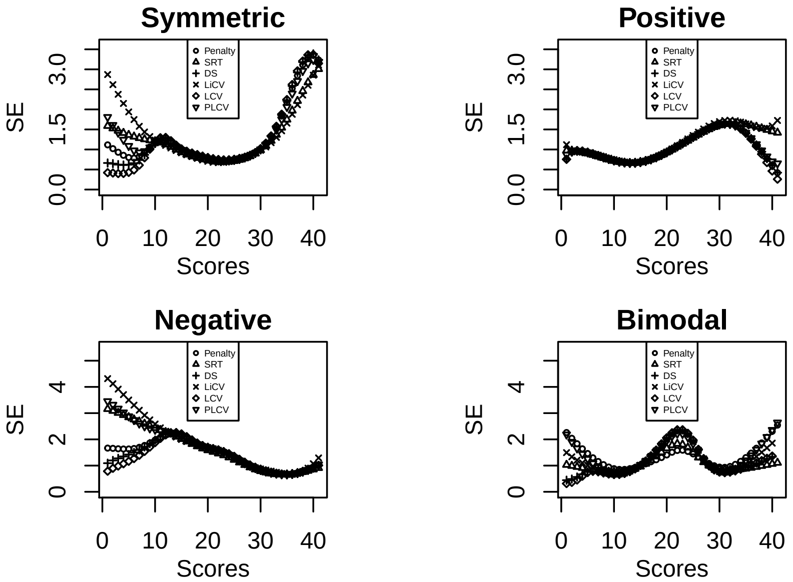

Figure C12: The SE under the NEAT design with a sample size of 100 and a test length of 40.

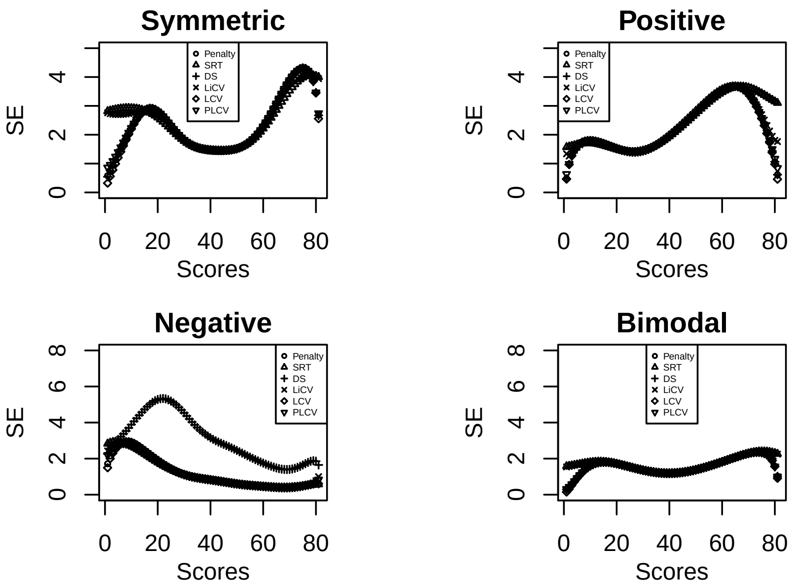

Figure C13: The SE under the NEAT design with a sample size of 100 and a test length of 80.

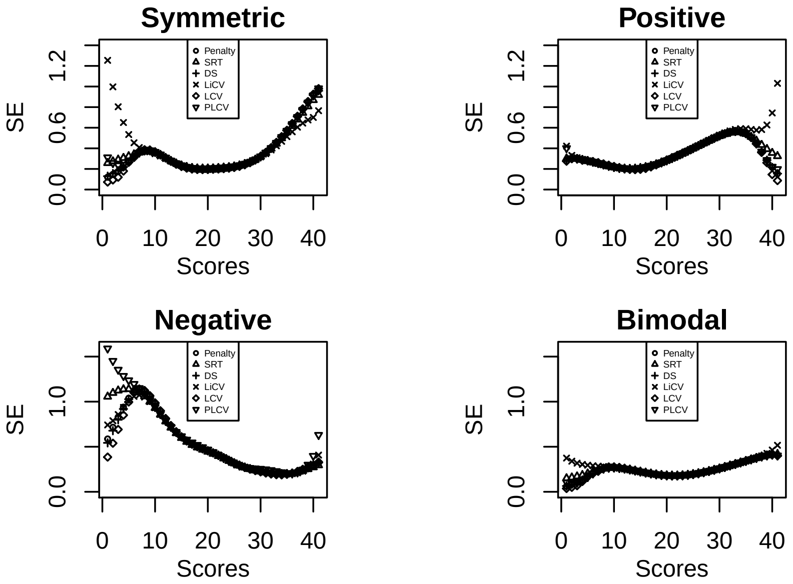

Figure C14: The SE under the NEAT design with a sample size of 1000 and a test length of 40.

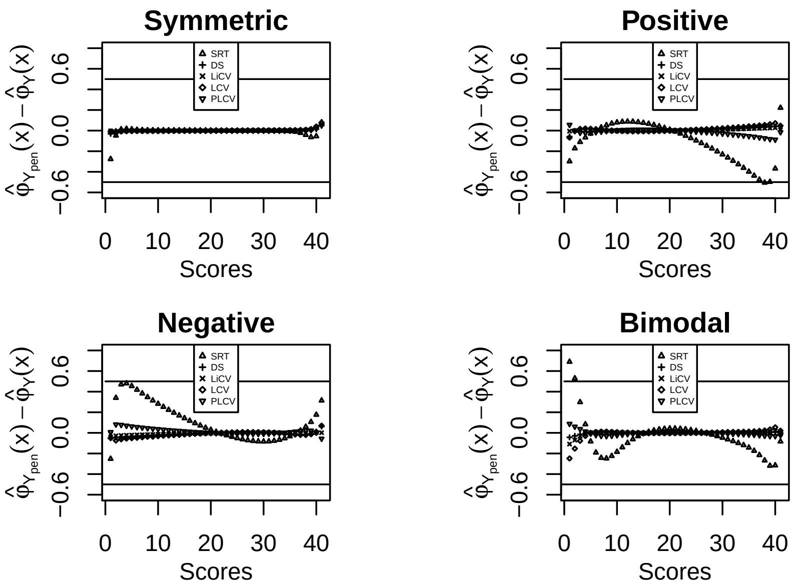

Figure C15: The mean difference to the KE estimator using the penalty method under the EG design, with a sample size of 100 and a test length of 40.

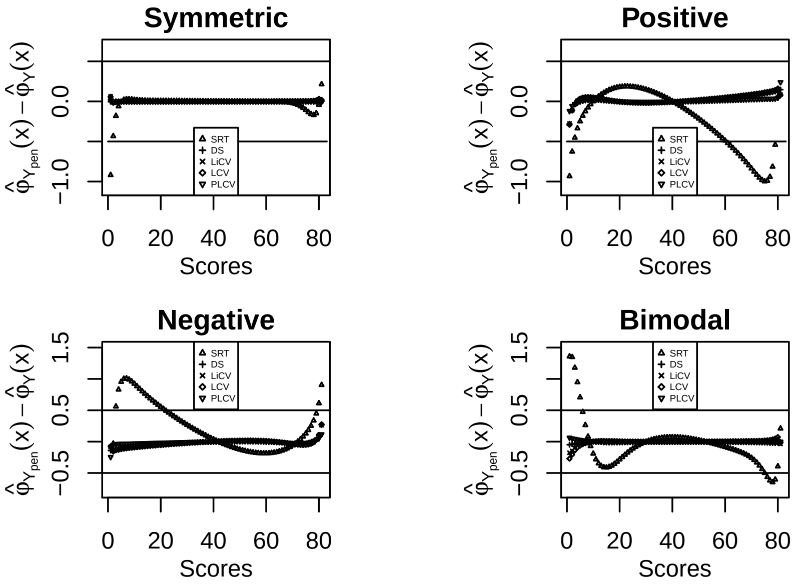

Figure C16: The mean difference to the KE estimator using the penalty method under the EG design, with a sample size of 100 and a test length of 80.

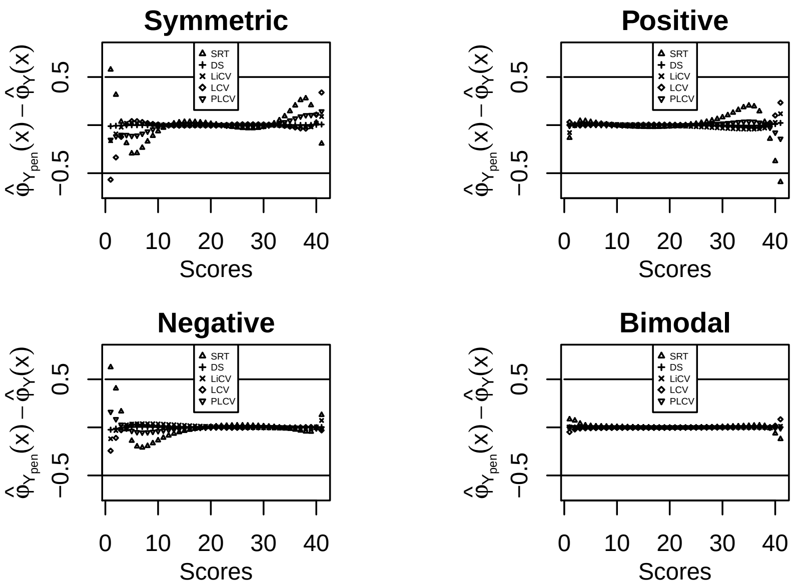

Figure C17: The mean difference to the KE estimator using the penalty method under the EG design, with a sample size of 1000 and a test length of 40.

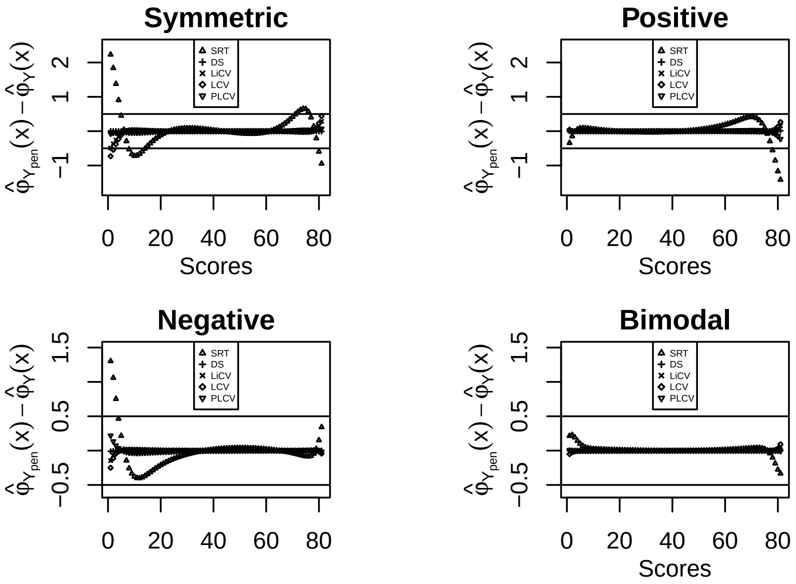

Figure C18: The mean difference to the KE estimator using the penalty method under the EG design, with a sample size of 1000 and a test length of 80.

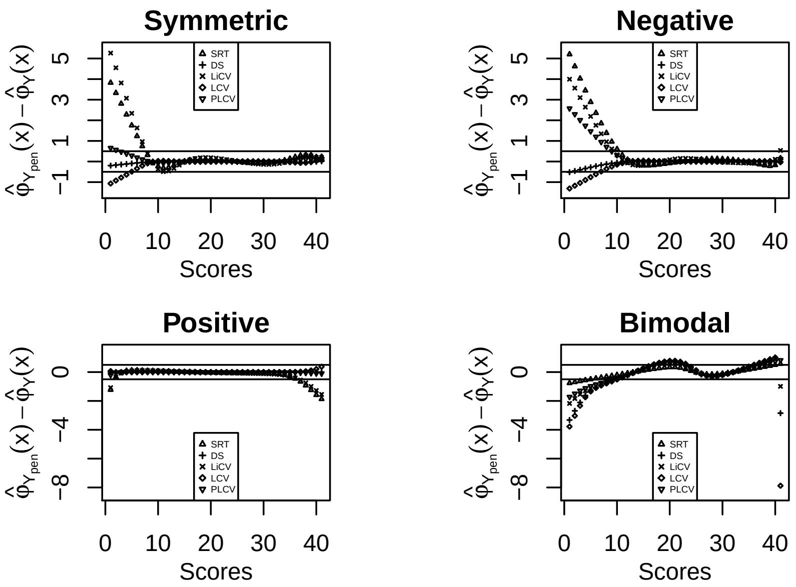

Figure C19: The mean difference to the KE estimator using the penalty method under the NEAT design, with a sample size of 100 and a test length of 40.

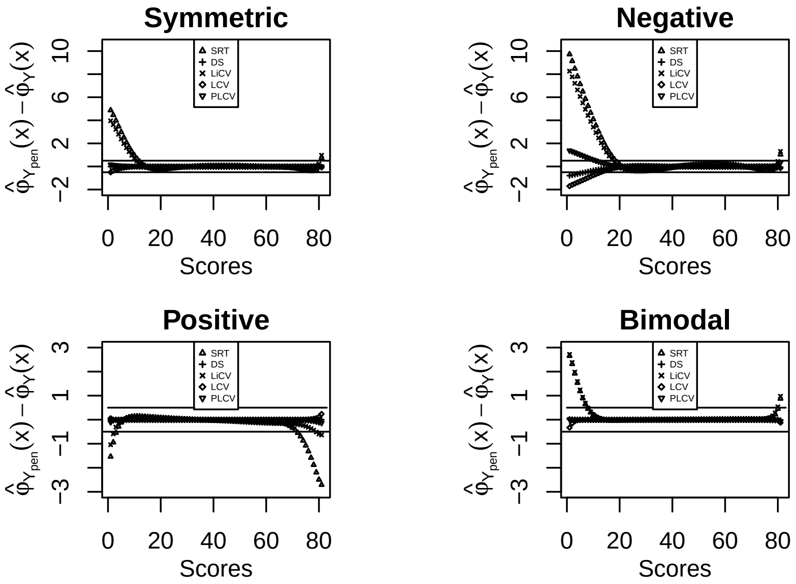

Figure C20: The mean difference to the KE estimator using the penalty method under the NEAT design, with a sample size of 100 and a test length of 80.

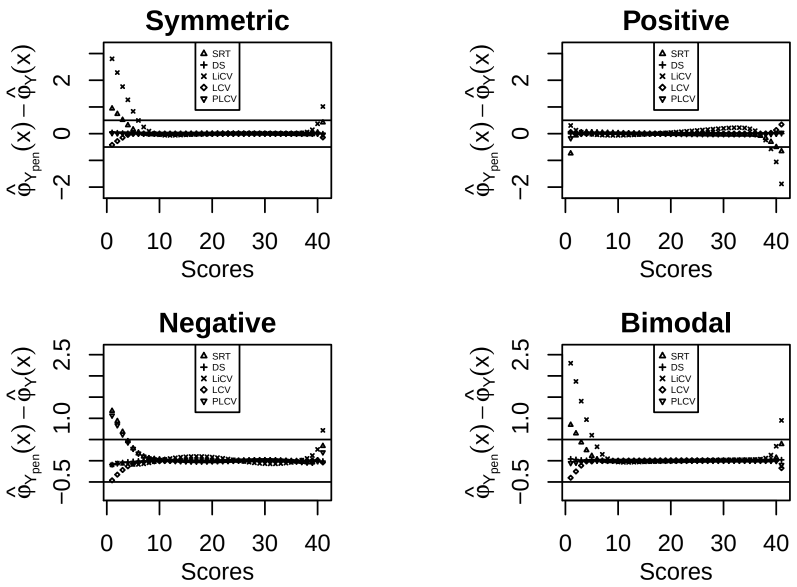

Figure C21: The mean difference to the KE estimator using the penalty method under the NEAT design, with a sample size of 1000 and a test length of 40.

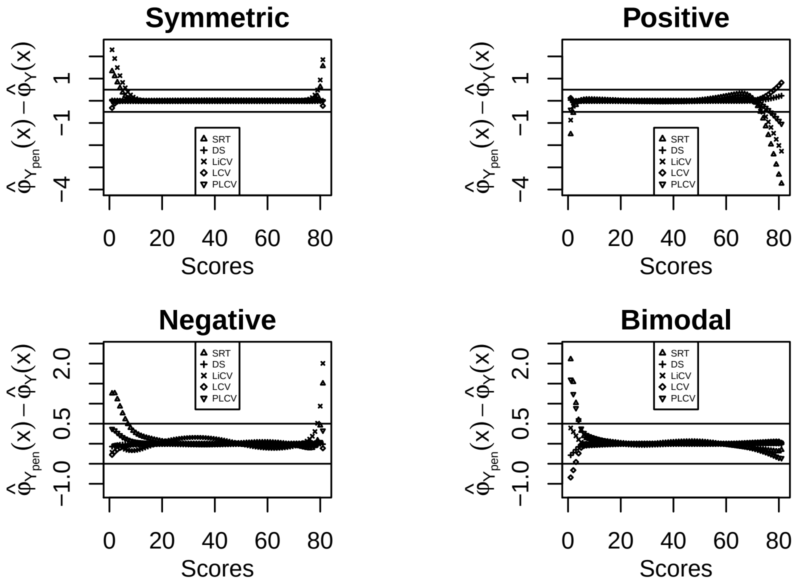

Figure C22: The mean difference to the KE estimator using the penalty method under the NEAT design, with a sample size of 1000 and a test length of 80.

Table D.1: The MSEs for all scenarios considered, under both the EG and NEAT design. The asterisk (*) indicates that the number of anchor items equals 40, otherwise they equal 20.

		EG						NEAT					
Distribution	Design	Penalty	SRT	DS	LiCV	LCV	PLCV	Penalty	SRT	DS	LiCV	LCV	PLCV
Symmetric	$n = 100, J-1 = 40$	0.765	0.765	0.765	0.764	0.765	0.765	0.329	0.326	0.328	0.326	0.329	0.327
	$n = 100, J-1 = 80$	2.891	2.894	2.892	2.891	2.892	2.891	1.638	1.624	1.638	1.627	1.638	1.637
	$n = 1000, J-1 = 40$	0.076	0.076	0.076	0.076	0.075	0.076	0.032	0.032	0.032	0.032	0.029	0.032
	$n = 1000, J-1 = 80$	0.288	0.287	0.288	0.288	0.288	0.288	0.111	0.111	0.111	0.111	0.111	0.111
	$n = 100, J-1 = 80*$							1.015	1.024	1.023	1.025	1.021	1.029
	$n = 1000, J-1 = 80*$							0.046	0.046	0.046	0.046	0.046	0.046
	$n = 5000, J-1 = 80*$							0.031	0.031	0.031	0.031	0.030	0.031
Pos. skewed	$n = 100, J-1 = 40$	0.779	0.779	0.778	0.779	0.778	0.778	0.412	0.411	0.412	0.412	0.412	0.412
	$n = 100, J-1 = 80$	3.206	3.207	3.206	3.206	3.206	3.206	1.732	1.733	1.732	1.735	1.733	1.733
	$n = 1000, J-1 = 40$	0.076	0.077	0.076	0.077	0.077	0.076	0.038	0.038	0.038	0.038	0.038	0.038
	$n = 1000, J-1 = 80$	0.302	0.303	0.302	0.302	0.303	0.302	0.151	0.147	0.151	0.150	0.152	0.150
	$n = 100, J-1 = 80*$							1.512	1.517	1.517	1.477	1.519	1.515
	$n = 1000, J-1 = 80*$							0.207	0.207	0.207	0.209	0.207	0.208
	$n = 5000, J-1 = 80*$							0.037	0.037	0.036	0.036	0.033	0.037
Neg. skewed	$n = 100, J-1 = 40$	0.745	0.746	0.745	0.745	0.745	0.745	0.495	0.492	0.495	0.490	0.495	0.494
	$n = 100, J-1 = 80$	3.189	3.185	3.189	3.189	3.189	3.189	1.889	1.874	1.889	1.875	1.889	1.889
	$n = 1000, J-1 = 40$	0.084	0.085	0.084	0.085	0.085	0.084	0.038	0.038	0.038	0.038	0.037	0.038
	$n = 1000, J-1 = 80$	0.301	0.302	0.301	0.301	0.301	0.301	0.561	0.550	0.561	0.552	0.562	0.561
	$n = 100, J-1 = 80*$							1.429	1.438	1.438	1.449	1.442	1.429
	$n = 1000, J-1 = 80*$							0.127	0.127	0.127	0.127	0.127	0.127
	$n = 5000, J-1 = 80*$							0.034	0.034	0.034	0.034	0.033	0.034
Bimodal	$n = 100, J-1 = 40$	0.665	0.665	0.665	0.665	0.665	0.665	0.891	0.882	0.948	0.934	0.950	0.914
	$n = 100, J-1 = 80$	2.742	2.737	2.742	2.742	2.742	2.742	1.412	1.409	1.412	1.408	1.411	1.412
	$n = 1000, J-1 = 40$	0.076	0.076	0.076	0.076	0.076	0.076	0.032	0.032	0.032	0.032	0.033	0.032
	$n = 1000, J-1 = 80$	0.285	0.285	0.285	0.285	0.285	0.285	0.347	0.329	0.347	0.347	0.348	0.344
	$n = 100, J-1 = 80*$							1.517	1.517	1.517	1.508	1.521	1.517
	$n = 1000, J-1 = 80*$							0.150	0.150	0.150	0.150	0.156	0.150
	$n = 5000, J-1 = 80*$							0.025	0.025	0.025	0.025	0.020	0.25
